# Structural Design and Control Research of Multi-Segmented Biomimetic Millipede Robot

**DOI:** 10.3390/biomimetics9050288

**Published:** 2024-05-11

**Authors:** Hao Yin, Ruiqi Shi, Jiang Liu

**Affiliations:** School of Mechanical and Automotive Engineering, Qingdao University of Technology, Qingdao 266520, China; yinhao_vehicle@qut.edu.cn (H.Y.); shiruiqi0351@163.com (R.S.)

**Keywords:** biomimetic robot, millipede, biologic, kinematic, CPG

## Abstract

Due to their advantages of good stability, adaptability, and flexibility, multi-legged robots are increasingly important in fields such as rescue, military, and healthcare. This study focuses on the millipede, a multi-segmented organism, and designs a novel multi-segment biomimetic robot based on an in-depth investigation of the millipede’s biological characteristics and locomotion mechanisms. Key leg joints of millipede locomotion are targeted, and a mathematical model of the biomimetic robot’s leg joint structure is established for kinematic analysis. Furthermore, a central pattern generator (CPG) control strategy is studied for multi-jointed biomimetic millipede robots. Inspired by the millipede’s neural system, a simplified single-loop CPG network model is constructed, reducing the number of oscillators from 48 to 16. Experimental trials are conducted using a prototype to test walking in a wave-like gait, walking with a leg removed, and walking on complex terrain. The results demonstrate that under CPG waveform input conditions, the robot can walk stably, and the impact of a leg failure on overall locomotion is acceptable, with minimal speed loss observed when walking on complex terrain. The research on the structure and motion control algorithms of multi-jointed biomimetic robots lays a technical foundation, expanding their potential applications in exploring unknown environments, rescue missions, agriculture, and other fields.

## 1. Introduction

With the advancement of technology, robotics plays an increasingly crucial role in various societal domains. As a significant branch, biomimetic legged robots, characterized by intermittent contact points with the ground and small contact areas, enable smooth robot movement. Their body, legs, and feet are arranged in parallel mechanisms, allowing them to adjust their posture to adapt to terrain requirements. Their efficient, flexible modes of operation and outstanding adaptability have made them a current research hotspot, possessing high scientific value and broad application prospects across multiple fields [1,2,3,4].

The research on multi-legged robots is limited, whether through simulation models or experimental prototypes. Inspired by common insects in the biological world, current biomimetic research on multi-legged robots mainly focuses on quadrupedal and hexapedal robots [5,6,7,8]. Although energy-efficient, their motion forms are limited, with poor load-bearing capacity and stability. In contrast, the biomimetic millipede robot, with its typical multi-legged structure, has a much larger number of legs than hexapods, featuring redundant support legs, strong load-bearing capacity, good stability, diverse gait combinations, high fault tolerance, and greater adaptability [9,10,11,12]. Currently, research on robots with more than six legs is relatively scarce, mainly focusing on single-joint structure design and driving methods. Wan et al. developed a leg mechanism controlled by a cam and driven by a single motor for legged robots [13], which does not require additional actuators during the leg returning phase. All legs can walk at a constant speed under constant rotational input. Garcia utilized a cam configuration to design a leg mechanism driven by a single electromagnetic DC motor [14]. Kano et al. developed a millipede-like robot, where each segment consists of a trunk and two legs [15], and its leg device is a single-motor-driven system with closed-loop feedback. Koh et al. proposed a modular robot inspired by millipedes [16], introducing the concept of a flexible body structure. The robot mimics the millipede’s elongated body, flexible body structure, and undulating gait using a single motor and flexible transmission shaft to achieve open-loop gait control and flexible body structure. Ozkan-Aydin et al. developed a low-cost hybrid multi-legged robot with eight parts [17], each with two limbs driven asynchronously. Both limb components and leg feet are driven by servo motors. Hoffman et al. developed a robot with multiple legs and a passive flexible backbone inspired by millipedes called millirobot [18], driven by piezoelectric bimorphs, requiring only two independent drive signals to perform various actions, demonstrating the advantages of multi-legged animal morphology in robot platform applications. Avirovik et al. developed a crawling robot driven by L-shaped piezoelectric actuators and a millipede-like robot driven by U-shaped piezoelectric actuators [19,20]. The motor structure consists of two piezoelectric bimorphs arranged in different configurations, causing elliptical motion at the end of the piezoelectric chip, driving the robot to move similarly to a millipede. The leg structures designed in the above studies are all driven by single motors and can only perform single-degree-of-freedom motion. They can achieve reciprocating motion of the legs through cam or linkage mechanisms. However, such structures cannot realize this motion on complex terrain and during multi-gait walking. Therefore, the three-joint, three-degrees-of-freedom leg structure designed in this paper addresses the problems of insufficient leg degrees of freedom and single control form in the above studies, achieving multi-degree-of-freedom leg motion and enabling more gait forms.

The central pattern generator (CPG) control method provides a natural and efficient motion control strategy for multi-legged robots, particularly suitable for simulating biological rhythmic movements, with promising applications in multi-legged robot motion control. Establishing a CPG control model and determining the appropriate CPG control model have become hot topics in current research. The concept of the motor neuron half-center model was first proposed by Thomas Graham Brown in 1912 [21], where two sets of mutually promoting and inhibiting spinal neurons could generate basic rhythmic movements. This theory reflects the concept of the widely accepted central pattern generator in today’s research. Matsuoka from Kyushu Institute of Technology proposed a mathematical discussion on sustained oscillations generated by mutual inhibition of neurons [22]. Kimura applied the CPG control model to the motion control of multi-legged robots [23]. His neural model consists of CPG, responses, and reflexes, where responses directly adjust the CPG phase rapidly, and reflexes directly produce joint torques. Righetti et al. developed a simple oscillator model that can independently adjust the duration of the rising and falling phases in the cycle [24], which is useful for independently adjusting swing and stance phases. They also proposed a new method of coupling two oscillators, which can use the theory of symmetrically coupled cells to construct the structure of the coupled oscillator network. Auke Jan Ijspeert proposed a spinal cord model and its implementation in the amphibious robot Salamander [25], demonstrating how to extend the primitive neural circuit for swimming by a system evolutionarily updated limb oscillation center to explain the salamander’s ability to switch between swimming and walking. Grabowska et al. built a model that can simulate the coordination patterns occurring in animals with eight legs (e.g., crayfish) and improve the model by modifying its original cyclic connection topology [26]. Zigen proposed a theoretical approach to build a new gait CPG controller for a quadruped locomotive based on delayed coupling VDP oscillators [27]. Lin et al. used a controller based on central pattern generators (CPGs) to coordinate the motion of the pectoral and caudal fins [28], achieving biomimetic motion of boxfish-like robots. While the above research utilizes CPG models to control specific robot movements, research on CPGs for millipede-style multi-legged robots is relatively scarce, and existing models are rather complex. Therefore, there is an urgent need to develop a CPG model suitable for millipede robots that is relatively simple.

**Figure 1:**
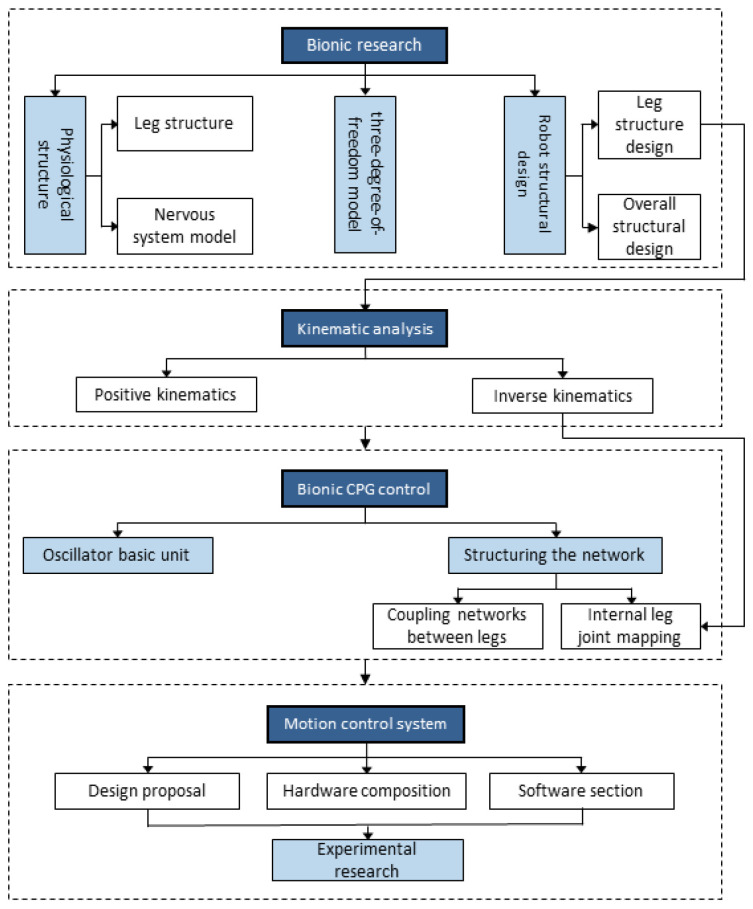
Technology roadmap.

This paper takes the millipede, a multi-segmented organism, as its research subject. From a biological perspective, it explores its neurological, skeletal, and arthropodal characteristics. Based on the leg configuration characteristics and neural architecture of millipedes, a novel multi-segment biomimetic robot is designed. Through structural optimization and an improved central pattern generator (CPG) control strategy, the aim is to achieve stable walking with high precision and large-scale motion in the biomimetic system inspired by millipedes. The technical roadmap of this paper is illustrated in Figure 1.

## 2. Biological Research

**Figure 2:**
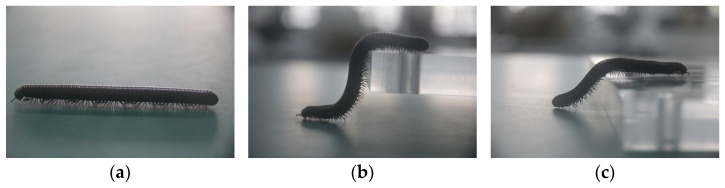
(**a**) Millipede walking on flat ground. (**b,c**) Morphology of millipedes when crossing barriers.

Millipedes possess elongated bodies and powerful legs, enabling them to move swiftly across complex terrain and effortlessly overcome obstacles. This biological structure grants them unique locomotive abilities, allowing adaptation to various terrains and environments, as illustrated in Figure 2. Compared to rigid-bodied insects, millipedes exhibit distinct advantages as multi-legged animals. First, due to their multiple legs, millipedes have numerous points of contact with the ground, providing enhanced stability. Second, their body structure grants them a high degree of flexibility. Their flexible spine allows them to adapt to the undulations of the terrain, maintaining close conformity to the terrain structure and enabling efficient movement. Additionally, millipedes demonstrate remarkable robustness. Even in the event of injury, they can adjust the gait of their other legs to maintain overall stability and swift movement.

**Figure 3:**
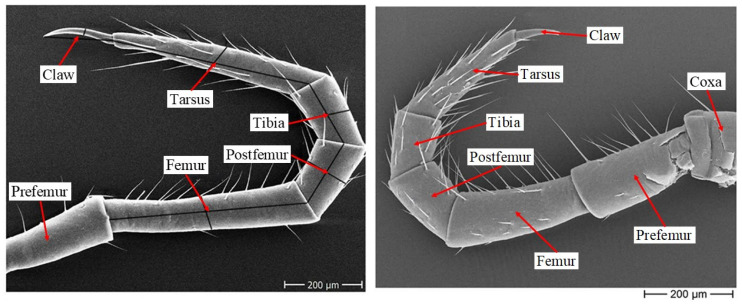
Model of the leg of a millipede.

The high stability, good flexibility, and strong robustness of millipedes stem from their multiple legs, which possess advantageous gaits and morphology. Therefore, it is important to first analyze their structure and gait characteristics. The leg structure of a millipede comprises seven parts: the coxa, prefemur, femur, postfemur, tibia, tarsus, and claw, as shown in Figure 3.

**Figure 4:**
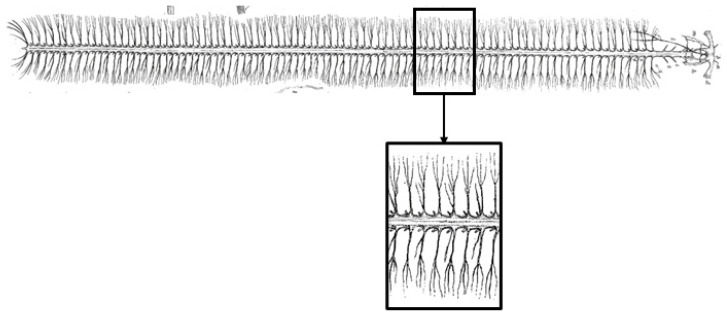
Diagram of the millipede nervous system.

The locomotion of a millipede is driven by muscles and controlled by the nervous system. Anatomical results are depicted in Figure 4. The central nervous system of a millipede consists of a brain, which transmits neural signals to various parts of the body via the ventral nerve cord. Each body segment is connected by paired nerves originating from large ganglia, emitting numerous small nerve branches on either side. Along the ventral nerve cord on both sides of the body are nerve plexuses called ganglia, with two ganglia per body segment. These ganglia then transmit neural signals to the muscle tissue within the corresponding body segments, driving and controlling the movement of the legs.

**Figure 5:**
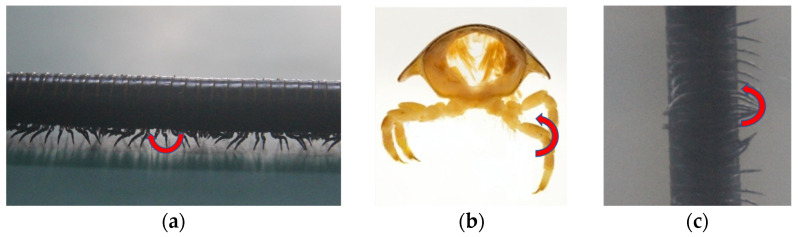
(**a**) Rotation around the vertical axis where the leg meets the body. (**b**) Rotation around an axis parallel to the body axis at the connection point. (**c**) About-body rotation on horizontal normal axis.

The gait of a millipede is determined by the movement of its walking legs, with each leg typically exhibiting three degrees of freedom, as shown in Figure 5. The first movement involves rotation around a vertical axis at the point where the leg connects to the body, allowing for swinging of the leg forward and backward (Figure 5a). The second movement enables each leg to rotate around an axis parallel to the body axis at the connection point, resulting in lifting motion of the leg (Figure 5b). The third movement involves rotation about the horizontal axis perpendicular to the body, causing the distal end of the leg to swing outward and bend (Figure 5c). Analyzing the movement of walking legs facilitates the structural design of biomimetic mechanisms.

The coordination of these three degrees of freedom enables the millipede robot to mimic the natural walking behavior of a millipede, achieving stable, efficient, and flexible locomotion. When designing the millipede robot, it is essential to fully consider the influence of these degrees of freedom and plan the structure and motion mechanism of the robot rationally to achieve optimal performance.

**Figure 6:**
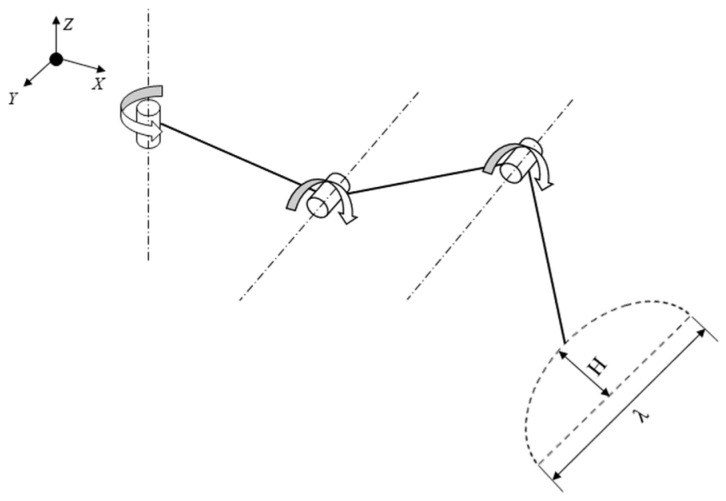
Three-degrees-of-freedom model.

Therefore, this paper proposes a three-degree-of-freedom model for the single leg motion of the millipede, as shown in Figure 6. The model is represented by the following equations:

(1) $$x(t)=\frac{\lambda }{2\pi t}\cos\left(\frac{2\pi t}{t_{sw}}\right)\;\;\;\;\;\;0\leq t\leq t_{sw},$$

(2) $$y(t)=\lambda \left[\frac{t}{t_{sw}}-\frac{1}{2\pi t}\sin\left(\frac{2\pi t}{t_{sw}}\right)\right]\;\;\;\;0\leq t\leq t_{sw},$$

(3) $$z(t)=\left\{\begin{array}{cc} 2H\left[\frac{t}{t_{sw}}-\frac{1}{4\pi }\sin\left(\frac{2\pi t}{t_{sw}}\right)\right] & 0\leq t\leq \frac{t_{sw}}{2}\\ 2H\left[1-\frac{2t}{t_{sw}}+\frac{1}{4\pi }\sin\left(\frac{4\pi t}{t_{sw}}\right)\right] & \frac{t_{sw}}{2}\leq t\leq t_{sw} \end{array}\right.,$$

where *λ* is the step length and *H* is the maximum leg lift height.

The three-degree-of-freedom model enables effective variation of the leg length from the driving end to the foot end, achieving stable vertical height of the trunk. Moreover, the spatial model is a more general model that through coordinated control of three coordinates, can encompass the planar model.

## 3. Structural Design and Mathematical Modeling

### 3.1. Single-Unit System

**Figure 7:**
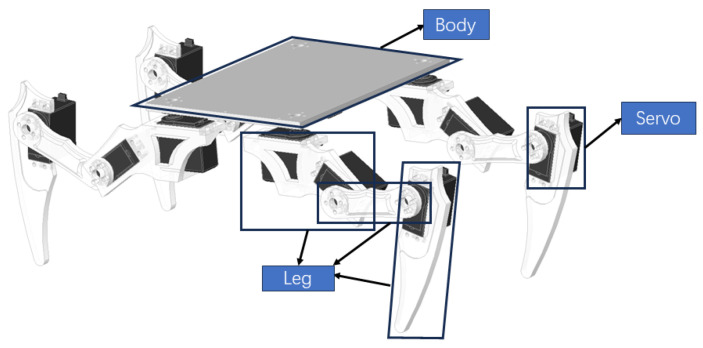
Single-unit model.

Based on the study of the morphological characteristics and gait of millipedes, we have successfully constructed a biomimetic millipede robot model. As discussed earlier, the body of a millipede consists of multiple segments, with each segment having four legs. Following this structure, we have designed a robot model composed of multiple modular systems interconnected with each other, with each modular system having four legs. This model is inspired by the biological features of millipedes and aims to simulate their mode of locomotion. Each modular unit is depicted in Figure 7.

**Figure 8:**
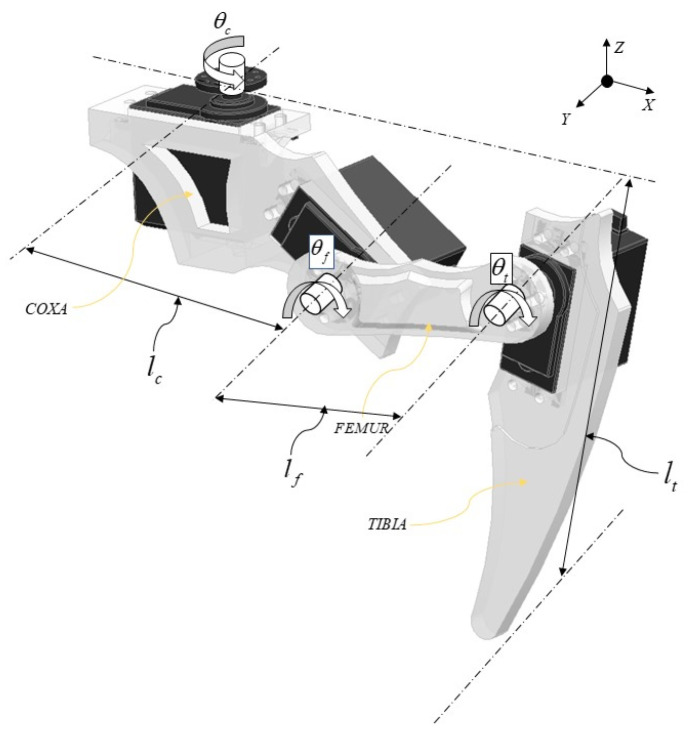
Leg model.

In our constructed simulation model, each leg of the millipede robot consists of a series mechanism composed of three servo motors, as illustrated in Figure 8. This configuration provides three degrees of freedom, corresponding to the three biological degrees of freedom shown in Figure 4. This design is inspired by an in-depth study of the biological characteristics and locomotion of millipedes, aiming to simulate the gaits and movements of millipedes more accurately in natural environments. The three degrees of freedom in the servo motor structure allow each leg to move flexibly in multiple directions, enabling the robot to efficiently adapt to complex terrains and environments. Each servo motor in the biomimetic millipede robot model is responsible for controlling the movement of the corresponding leg joint, mimicking the actions of millipede legs during ground locomotion.

### 3.2. Kinematics

The movement of the swing leg is analogous to a serial manipulator structure, with the body acting as the support. Therefore, we will proceed with the kinematic analysis of the swing leg. Forward kinematics refers to determining the posture and position of the swing leg end when the joint angles are known.

**Figure 9:**
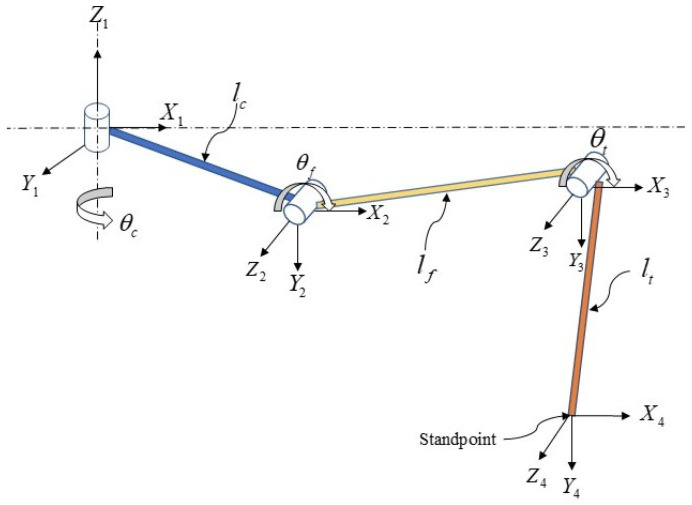
Kinematic modeling of the single leg.

According to the biological research outlined earlier, we know that a millipede’s leg comprises seven parts. In the robot designed in this paper, we simplify the leg structure to three parts: the hip, femur, and tibia. The connection between the body and the hip joint is denoted by *θ*_c_, the connection between the hip and femur joint is denoted by *θ*_f_, and the connection between the femur and tibia joint is denoted by *θ*_t_, as shown in Figure 9. In this paper, we employ the Denavit–Hartenberg (D-H) method for the kinematic analysis of the robot’s walking leg [29], representing the position of the foot relative to the body and the hip joint’s rotation axis as [px py pz]^T^. The D-H parameters for the leg are as shown in Table 1.

The coordinate transformation equation between adjacent joints is given by (4).

(4) T40=cosθccosθf+θt−cosθcsinθf+θtsinθccosθclc+lfcosθf+ltcos(θf+θt)sinθccosθf+θt−sinθccosθf+θt−cosθcsinθclc+lfcosθf+ltcos(θf+θt)sinθf+θtcosθf+θt0ltsinθf+θt+lfsinθf0001

Then, the position of the foot end is given by (5).

(5) $$\left[\begin{array}{c} p_{x}\\ p_{y}\\ p_{z} \end{array}\right]=\left[\begin{array}{c} \cos\theta_{c}\left(l_{c}+l_{f}\cos\theta_{f}+l_{t}\cos(\theta_{f}+\theta_{t})\right)\\ \sin\theta_{c}\left(l_{c}+l_{f}\cos\theta_{f}+l_{t}\cos(\theta_{f}+\theta_{t})\right)\\ l_{t}\sin\left(\theta_{f}+\theta_{t}\right)+l_{f}\sin\theta_{f} \end{array}\right]$$

The structural analysis conducted through the aforementioned forward kinematics calculation indicates that the robot’s design meets the requirements, laying the foundation for the subsequent development of motion control theory for the biomimetic millipede robot.

### 3.3. Inverse Kinematics

To achieve precise control of the joint angles during the robot’s motion, it is necessary to perform inverse kinematic analysis of the walking leg. This paper employs algebraic methods to conduct inverse kinematic analysis of the walking leg.

The known stance and position of the foot end of the walking foot is shown in Equation (6).

(6) $$\begin{array}{l} \;\left[\begin{array}{cccc} \cos\theta_{c}n_{x}+\sin\theta_{c}n_{y} & \cos\theta_{c}o_{x}+\sin\theta_{c}o_{y} & \cos\theta_{c}a_{x}+\sin\theta_{c}a_{y} & \cos\theta_{c}p_{x}+\sin\theta_{c}p_{y}\\ -\sin\theta_{c}n_{x}+\cos\theta_{c}n_{y} & -\sin\theta_{c}o_{x}+\cos\theta_{c}o_{y} & -\sin\theta_{c}a_{x}+\cos\theta_{c}a_{y} & -\sin\theta_{c}p_{x}+\cos\theta_{c}p_{y}\\ n_{z} & o_{z} & a_{z} & p_{z}\\ 0 & 0 & 0 & 1 \end{array}\right]\\ =\left[\begin{array}{cccc} \cos\left(\theta_{f}+\theta_{t}\right) & -\sin\left(\theta_{f}+\theta_{t}\right) & 0 & l_{t}\cos\left(\theta_{f}+\theta_{t}\right)+l_{f}\cos\theta_{f}+l_{c}\\ 0 & 0 & -1 & 0\\ \sin\left(\theta_{f}+\theta_{t}\right) & \cos\left(\theta_{f}+\theta_{t}\right) & 0 & l_{t}\sin\left(\theta_{f}+\theta_{t}\right)+l_{f}\sin\theta_{f}\\ 0 & 0 & 0 & 1 \end{array}\right] \end{array}$$

Comparing r_24_ elements of both sides of the equation yields (7).

(7) $$-\sin\theta_{c}p_{x}+\cos\theta_{c}p_{y}=0$$

The joint angle *θ*_c_ can be expressed as (8).

(8) $$\theta_{c}=\arctan\left(\frac{p_{y}}{p_{x}}\right)$$

We calculate *θ*_f_ and *θ*_t_ via (9).

(9) $$\begin{array}{l} \left[\begin{array}{cccc} f_{1}(n) & f_{1}(o) & f_{1}(a) & \cos\theta_{f}\left(\cos\theta_{c}p_{x}+\sin\theta_{c}p_{y}\right)+\sin\theta_{f}p_{z}-l_{c}c_{2}\\ f_{2}(n) & f_{2}(o) & f_{2}(a) & -\sin\theta_{f}\left(\cos\theta_{c}p_{x}+\sin\theta_{c}p_{y}\right)+\cos\theta_{f}p_{z}+l_{c}s_{2}\\ f_{3}(n) & f_{3}(o) & f_{3}(a) & \sin\theta_{c}p_{x}-\cos\theta_{c}p_{y}\\ 0 & 0 & 0 & 1 \end{array}\right]\\ =\left[\begin{array}{cccc} \cos\theta_{t} & -\sin\theta_{t} & 0 & l_{t}\cos\theta_{t}+l_{f}\\ \sin\theta_{t} & \cos\theta_{t} & 0 & l_{t}\sin\theta_{t}\\ 0 & 0 & 1 & 0\\ 0 & 0 & 0 & 1 \end{array}\right] \end{array}$$

Comparing r_14_ and r_24_ elements of both sides of the equation yields (10).

(10) $$\left\{\begin{array}{l} \cos\theta_{f}\left(\cos\theta_{c}p_{x}+\sin\theta_{c}p_{y}-l_{c}\right)+\sin\theta_{f}p_{z}=l_{t}\cos\theta_{t}+l_{f}\\ -\sin\theta_{f}\left(\cos\theta_{c}p_{x}+\sin\theta_{c}p_{y}\right)+\cos\theta_{f}p_{z}+l_{c}\sin\theta_{f}=l_{t}\sin\theta_{t} \end{array}\right.$$

This solves the following:

(11) $$\theta_{f}=\arccos\frac{p_{z}}{\sqrt{{\left(\cos\theta_{c}p_{x}+\sin\theta_{c}p_{y}-l_{c}\right)}^{2}+p_{z}^{2}}}-\arcsin\left[\frac{{\left(\cos\theta_{c}p_{x}+\sin\theta_{c}p_{y}-l_{c}\right)}^{2}+p_{z}^{2}+l_{f}^{2}-l_{t}^{2}}{\sqrt{4l_{f}^{2}\left({\left(\cos\theta_{c}p_{x}+\sin\theta_{c}p_{y}-l_{c}\right)}^{2}+p_{z}^{2}\right)}}\right]$$

(12) $$\theta_{t}=-\arccos\left[\frac{\left(\cos\theta_{c}p_{x}+\sin\theta_{c}p_{y}-l_{c}\right)\cos\theta_{f}+p_{z}\sin\theta_{f}-l_{f}}{l_{t}}\right].$$

## 4. Improved CPG Control Algorithm

CPG is a biologically inspired control method that mimics the neural networks responsible for generating basic movement patterns in the central nervous system. It drives robot motion by autonomously generating periodic signals without the need for external cyclic inputs. CPGs are capable of producing stable oscillatory behavior automatically in the absence of higher-level control signals and external feedback, while higher-level signals and external feedback can modulate and stabilize CPGs [30,31,32].

### 4.1. CPG Oscillation Unit Model

In this paper, the Hopf oscillator is chosen as the unit model of CPG, and its mathematical model is as follows [33]:

(13) $$\left\{\begin{array}{c} \dot{x}=\alpha \left(\mu -r^{2}\right)x-\omega y\\ \dot{y}=\beta \left(\mu -r^{2}\right)y+\omega x\\ \omega =\frac{\omega_{\mathrm{st}}}{e^{-\mathrm{ay}}+1}+\frac{\omega_{\mathrm{sw}}}{e^{ay}+1}\\ \omega_{\mathrm{st}}=\frac{1-\beta }{\beta }\omega_{\mathrm{sw}} \end{array}\right.,$$

where *x* and *y* denote state variables of the oscillator; $r^{2}={\left(x-u_{1}\right)}^{2}+{\left(y-u_{2}\right)}^{2}$ is the amplitude of the oscillator output signal; $\sqrt{u}$ is the frequency of the oscillator; $\omega_{sw\;}$ is the frequency of the swing phase; $\omega_{st}$ is the frequency of the support phase; and *β* is the duty cycle.

### 4.2. CPG Control Network

**Figure 10:**
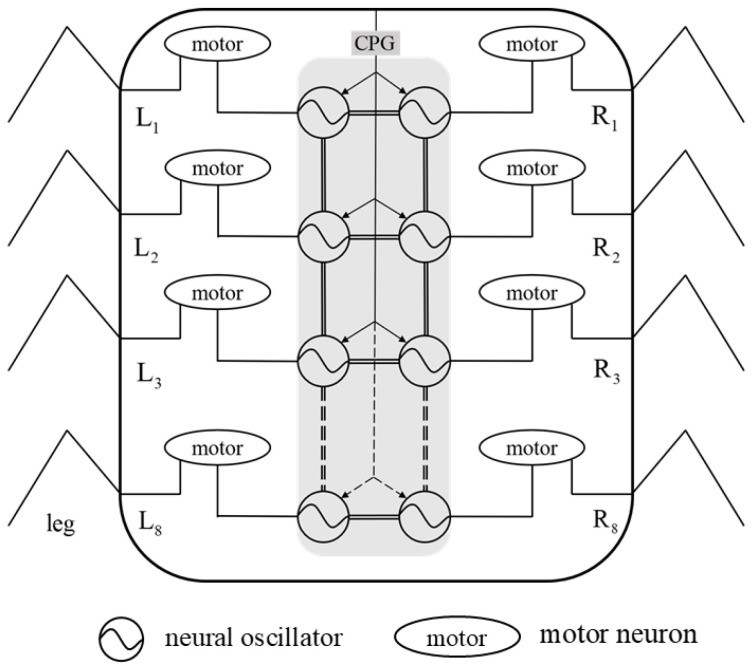
CPG control network model.

In traditional CPG-based robot gait control strategies, the CPG model is solely applied in the temporal domain, meaning it is only used to achieve inter-leg coordination of the robot without considering the coordination among joints within a single leg. Therefore, the coupling characteristics of biological CPGs are not fully utilized. This section will investigate a method whereby CPGs are simultaneously utilized to achieve coordination control of both inter-leg and intra-leg joints in millipede-like robots, expanding the application of CPGs from the temporal domain to the spatial domain. Combining with the neural system structure diagram of the millipede shown in Figure 4, this paper proposes an improved CPG network coupling model. In this model, each leg only requires the first joint to utilize an independent CPG oscillator to generate the joint angle output signal, while the second and third joints generate joint angles through mapping functions. With this improvement, the entire CPG network model only needs 16 oscillators to form a complete CPG network, reducing the complexity of the system. The CPG network connection methods are divided into circular coupling structure and fully symmetrical coupling structure. Since the walking legs of the millipede robot in this paper are symmetrically distributed on both sides of the body, they are divided into two groups, and there is no interference between the walking legs of the two groups. Therefore, the single-ring network’s inter-leg coupling structure is more suitable for the gait control of the robot in this paper. In summary, the improved CPG network model designed in this paper is illustrated in Figure 10.

#### 4.2.1. Interfoot Coordination Control

Based on the neural structural relationship of the millipede depicted in Figure 4, the current study establishes a framework for inter-leg coordination of the millipede robot. This is achieved by establishing mutual coupling relationships among 16 hip joint oscillators (corresponding to the bilateral 16 nerve cords in Figure 4’s neural system diagram). This is illustrated in Figure 10.

The mathematical model of the CPG network consisting of 16 Hopf oscillators is

(14) $$\left\{\begin{array}{c} \left[\begin{array}{c} {\dot{x}}_{i}\\ {\dot{y}}_{i} \end{array}\right]=\left[\begin{array}{cc} \alpha \left(\mu -r_{i}^{2}\right) & -\omega_{i}\\ \omega_{i} & \alpha \left(\mu -{r_{i}}^{2}\right) \end{array}\right]\left[\begin{array}{c} x_{i}\\ y_{i} \end{array}\right]+\sum_{j=1}^{16}R\left(\theta_{i}^{j}\right)\left[\begin{array}{c} x_{j}\\ y_{j} \end{array}\right],i=1,\cdots ,16\\ r_{i}^{2}=x_{i}^{2}+y_{i}^{2}\\ \omega_{i}=\frac{\omega_{\mathrm{st}}}{e^{-ay_{i}}+1}+\frac{\omega_{\mathrm{sw}}}{e^{ay_{i}}+1}\\ \omega_{\mathrm{st}}=\frac{1-\beta }{\beta }\omega_{\mathrm{sw}} \end{array}\right.,$$

where *x_i_* is the output of the oscillator, used as the hip joint angle control signal; that is, $\theta_{hi}=x_{i}$; the second term on the right side represents the coupling between oscillators; $\theta_{i}^{j}$ denotes the relative phase between oscillators *i* and *j*; and $R(\theta_{i}^{j})$ is the rotation matrix, which characterizes the phase coupling relationship between different oscillators. Its expression is as follows:

(15) $$R_{ji}=R\left(\theta_{j}^{i}\right)=\left[\begin{array}{cc} y_{j} & \sin\theta_{j}^{i}\\ x_{j} & \cos\theta_{j}^{i} \end{array}\right].$$

According to the coupling mathematical model of the multi-foot oscillator of (14), the following mathematical expression for the toroidal coupling relationship of the 16 hip Hopf oscillator is obtained:

(16) $$\left[\begin{array}{c} {\dot{x}}_{1}\\ \vdots \\ {\dot{x}}_{16} \end{array}\right]=-\alpha \left[\begin{array}{ccc} x_{1}^{2}+y_{1}^{2}-1 & \cdots & 0\\ \vdots & \ddots & \vdots \\ 0 & \cdots & x_{16}^{2}+y_{16}^{2}-1 \end{array}\right]\left[\begin{array}{c} x_{1}\\ \vdots \\ x_{16} \end{array}\right]-\left[\begin{array}{ccc} \omega_{1} & \cdots & 0\\ \vdots & \ddots & \vdots \\ 0 & \cdots & \omega_{16} \end{array}\right]\left[\begin{array}{c} y_{1}\\ \vdots \\ y_{16} \end{array}\right]+\delta \cdot G\left[\begin{array}{c} y_{1}\\ \vdots \\ y_{16} \end{array}\right]-\delta \cdot M\left[\begin{array}{c} x_{1}\\ \vdots \\ x_{16} \end{array}\right],$$

(17) $$\left[\begin{array}{c} {\dot{y}}_{1}\\ \vdots \\ {\dot{y}}_{16} \end{array}\right]=-\alpha \left[\begin{array}{ccc} x_{1}^{2}+y_{1}^{2}-1 & \cdots & 0\\ \vdots & \ddots & \vdots \\ 0 & \cdots & x_{16}^{2}+y_{16}^{2}-1 \end{array}\right]\left[\begin{array}{c} y_{1}\\ \vdots \\ y_{16} \end{array}\right]+\left[\begin{array}{ccc} \omega_{1} & \cdots & 0\\ \vdots & \ddots & \vdots \\ 0 & \cdots & \omega_{16} \end{array}\right]\left[\begin{array}{c} x_{1}\\ \vdots \\ x_{16} \end{array}\right]+\delta \cdot G\left[\begin{array}{c} y_{1}\\ \vdots \\ y_{16} \end{array}\right]-\delta \cdot M\left[\begin{array}{c} x_{1}\\ \vdots \\ x_{16} \end{array}\right],$$

(18) $$\omega_{i}=\frac{\omega_{\mathrm{st}}}{e^{-ax_{i}}+1}+\frac{\omega_{\mathrm{sw}}}{e^{ax_{i}}+1}(i=1,2,\cdots ,16),$$

where ***G*** and ***M*** are matrix coupling coefficients, and if the oscillators are uncorrelated with each other, then the coefficients of the corresponding terms are zero.

(19) $$G=\left[\begin{array}{ccccc} \cos\theta_{11} & \cdots & \cdots & \cdots & \cos\theta_{16\;1}\\ \vdots & \ddots & \cdots & \cdots & \vdots \\ \vdots & \cdots & \cos\theta_{88} & \cdots & \vdots \\ \vdots & \cdots & \cdots & \ddots & \vdots \\ \cos\theta_{1\;16} & \cdots & \cdots & \cdots & \cos_{16\;16} \end{array}\right]$$

(20) $$M=\left[\begin{array}{ccccc} \sin\theta_{11} & \cdots & \cdots & \cdots & \sin\theta_{16\;1}\\ \vdots & \ddots & \cdots & \cdots & \vdots \\ \vdots & \cdots & \sin\theta_{88} & \cdots & \vdots \\ \vdots & \cdots & \cdots & \ddots & \vdots \\ \sin\theta_{1\;16} & \cdots & \cdots & \cdots & \sin_{16\;16} \end{array}\right]$$

When the millipede robot walks using a wave-like gait, the phase difference between legs on each side is π/4, and the phase difference between each pair of legs is π. This arrangement allows the legs on both sides of the robot to swing cyclically with the same phase difference, thereby completing the entire gait cycle.

#### 4.2.2. Intrafoot Coordination Control

**Figure 11:**
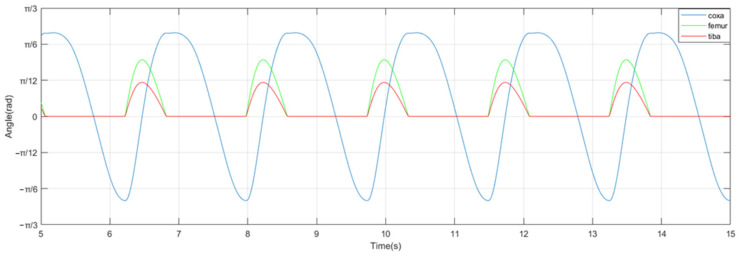
Output curves of each joint within the leg in traveling wave gait.

The CPG network is responsible for generating periodic control signals for each joint. However, controlling all joints of the robot using CPG networks would significantly increase the number of output periodic signals, thus increasing the complexity of the entire CPG network and reducing real-time performance due to prolonged computation time. To address this issue, CPG control can be applied only to the hip joints of the biomimetic millipede robot. Then, through the mapping relationship between the femur and tibia joints with the hip joint, the motion of the femur and tibia joints of the same walking leg can be controlled. This significantly reduces the number of required control signals, simplifying the overall CPG network structure of the millipede robot. Based on this, the following control scheme is proposed: using 16 Hopf oscillators corresponding to the 16 legs of the millipede robot, the x output of the oscillators is directly used as the angle control signal for the hip joints, while the y output is transformed and then used as the angle control signal for the femur and tibia joints. The expression for the control signal of a single leg is as follows:

(21) $$\left\{\begin{array}{c} \theta_{hi}=x_{i}\\ \theta_{fi}=\left\{\begin{array}{c} -\mathrm{sgn}(\varphi )\frac{A_{f}}{A_{h}}y_{i},y_{i}\leq 0\\ 0,y_{i}>0 \end{array}\right.\\ \theta_{ti}=\left\{\begin{array}{c} -\mathrm{sgn}(\varphi )\frac{A_{t}}{A_{h}}y_{i},y_{i}\leq 0\\ 0,y_{i}>0 \end{array}\right. \end{array}\right.$$

where *A_h_*, *A_f_*, and *A_t_* are the hip, femur, and tibia joint amplitudes, respectively. The output curves of each joint within the leg of the millipede robot traveling wave gait can be generated as shown in Figure 11.

## 5. Robot Physical Prototypes and Experiments

In this section, a physical prototype of the millipede robot will be constructed based on the design outlined in the previous sections. Subsequently, a motion control system will be designed according to the neural structure diagram of the millipede. Finally, experiments will be conducted to validate the feasibility of the aforementioned structure and the CPG control model.

### 5.1. Robot Hardware Composition

**Figure 12:**
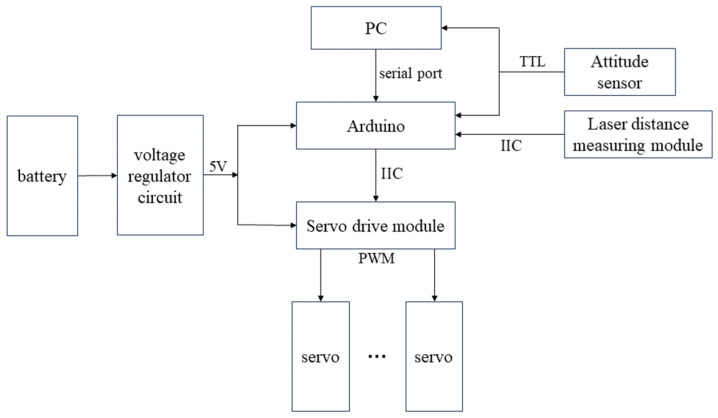
Hardware block diagram of motion control system for millipede robot.

The hardware composition structure of the bio-inspired millipede robot’s motion control system is shown in Figure 12. The core controller of the system adopts an Arduino Uno control board, which is responsible for the overall motion control logic and data processing of the robot. In addition, the system includes servo motor driver modules, TOF laser ranging modules, a power supply module, and an attitude sensor. The workflow of the system is as follows: The CPG model is discretized to generate the gait data required for the leg movements of the robot. These data are converted into specific servo motor rotation commands within the controller (Arduino). These commands are transmitted to the servo motor driver modules via I2C communication. Upon receiving the commands, the servo motor driver modules generate corresponding drive signals to control the servo motors of the robot’s legs. The servo motors precisely execute the leg movements according to the gait test data, thereby driving the robot to perform coordinated and stable movements. During the robot’s motion, the attitude sensor (MPU6050) continuously monitors the robot’s posture, while the TOF laser ranging sensor measures the distance between the robot and surrounding obstacles in real-time. If the measured distance falls below a predetermined safety threshold, indicating a potential collision with obstacles, the robot immediately pauses its motion to ensure its safety.

**Figure 13:**
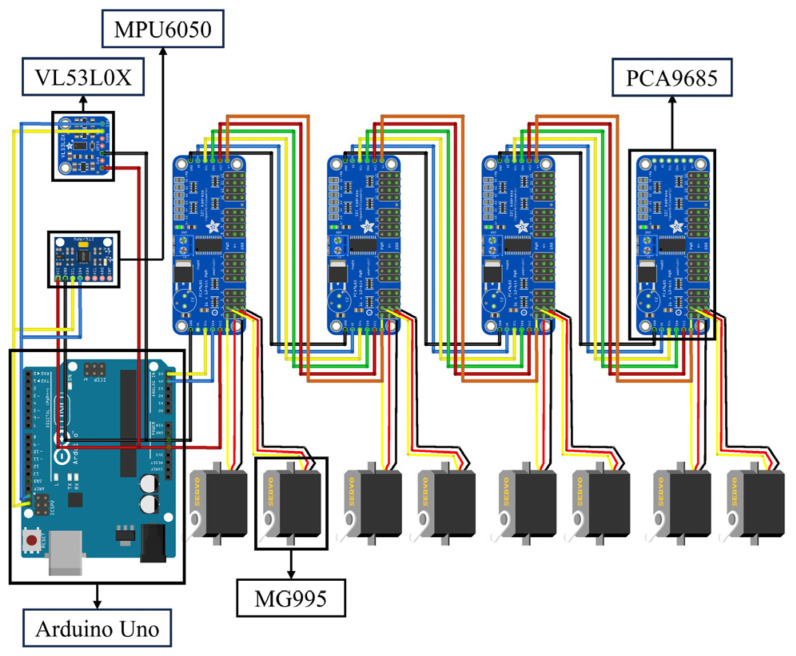
Robot motion control system hardware connection diagram.

To control the rotation of 48 servos, four PCA9685 control boards are connected in series, with each PCA9685 board capable of controlling 12 servos. These boards are then connected to an Arduino Uno to form the entire control system. The hardware connection diagram is shown in Figure 13.

Each PCA9685 board has a set of address jumpers in the upper right corner, allowing users to change the communication address of the board by shorting certain points. This design enables a single Arduino to conveniently control multiple servo control boards without communication conflicts. By using different jumper combinations, a unique address can be created for up to 62 servo control boards operating simultaneously. In this system, the addresses of the four PCA9685 boards are set to 0 × 40, 0 × 41, 0 × 42, and 0 × 43, respectively. If it is necessary to change the address of a board, we simply solder a jumper on the two address terminals. Additionally, each PCA9685 servo control board is equipped with two power terminals at the top, requiring a power supply of 5–6 V. These power terminals not only provide power to the control board itself but also supply the required power to the 16 output terminals at the bottom for controlling the servos.

### 5.2. Robot Software Section

**Figure 14:**
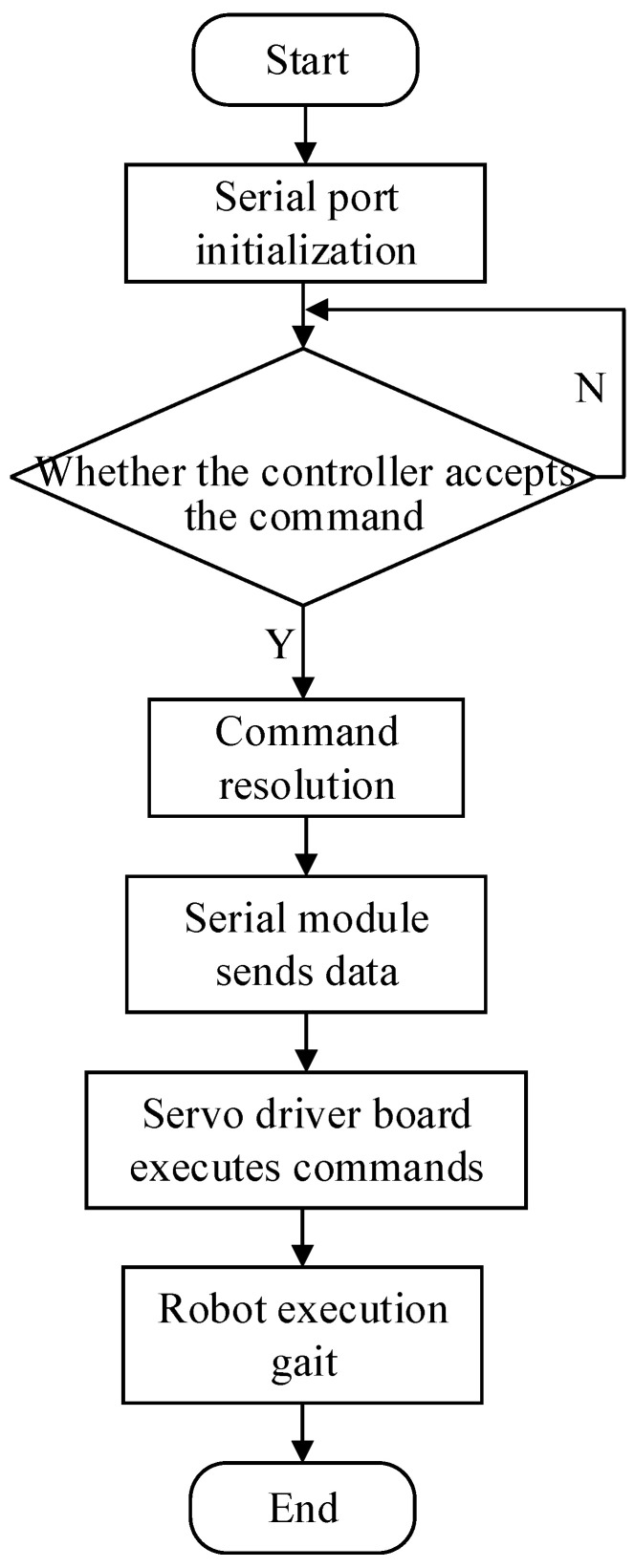
Flowchart for motion control of bionic millipede robot.

The software part of the biomimetic millipede robot’s motion control system is tasked with converting the designed CPG control model from previous chapters into code suitable for execution on the Arduino platform. This process involves translating complex mathematical models and algorithms into concise instructions that Arduino can understand and execute. By writing and compiling code in the Arduino IDE, the code is downloaded into the Arduino Uno microcontroller. The motion control flowchart of the biomimetic millipede robot is illustrated in Figure 14.

### 5.3. Experimental

**Figure 15:**
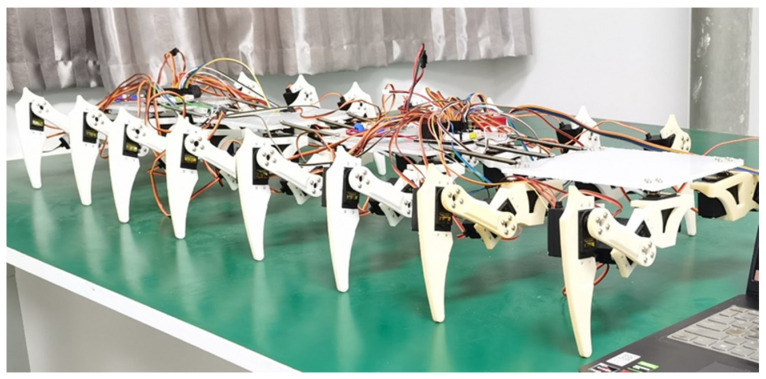
Physical prototype of the bionic millipede robot.

After modeling the structural design from Section 3 and 3D printing the components, the physical prototype of the millipede robot is assembled using screws, as shown in Figure 15. The biomimetic millipede robot prototype consists primarily of the main body trunk and sixteen legs. The body segments are connected by springs, and the leg joints are driven by servo motors. As mentioned earlier, the body segments of the robot are rectangular in shape, facilitating the installation of motion control systems, power modules, and other components. The legs of the robot are arranged uniformly around the body trunk for balanced distribution.

The main structural parameters of the bionic millipede robot prototype are shown in Table 2.

#### 5.3.1. Standard Traveling Wave Gait Walking

The output signals of the traveling wave gait CPG model are utilized to construct a ring-like network of CPG coupling structure to ensure stable phase differences among the robot’s legs. Building upon this, a mapping function for the internal joints of the robot legs is further established. This function is capable of outputting the rotational angle values of the hip joint, femur joint, and tibia joint within a single leg of the robot. These output angle values undergo discretization processing and are then used in gait validation experiments to verify the robot’s gait performance during actual motion.

**Figure 16:**
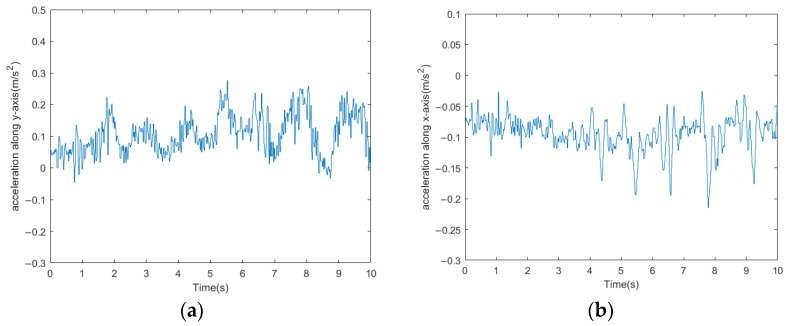
(**a**) Longitudinal acceleration profiles of walking with traveling wave gait in a bionic millipede robot. (**b**) Walking transverse acceleration profile of a bionic millipede robot with traveling wave gait.

**Figure 17:**
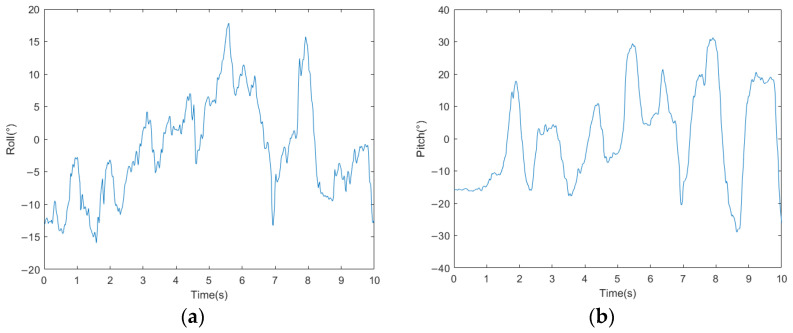
(**a**) Bionic millipede robot traveling wave gait *x*-axis attitude angle profile. (**b**) Bionic millipede robot traveling wave gait *y*-axis attitude angle profile.

The acceleration curves of the bio-inspired millipede robot’s traveling wave gait are depicted in Figure 16. From the figure, it can be observed that the fluctuation of longitudinal acceleration is less than 0.2 m/s^2^, and the fluctuation of lateral acceleration is less than 0.3 m/s^2^. The experimental results indicate that the bio-inspired millipede robot exhibits smooth motion and coordinated gait during walking. The pitch attitude angles of the robot platform during the traveling wave gait are illustrated in Figure 17. Since the rotation around the *z*-axis does not affect the stability of the robot’s walking, the angle variation curve is not presented. However, from the curves of the *x*-axis and *y*-axis attitude angles, it can be inferred that the bio-inspired millipede robot possesses a good longitudinal straight-line walking capability with minimal attitude angles, ensuring stability throughout the walking process.

**Figure 18:**
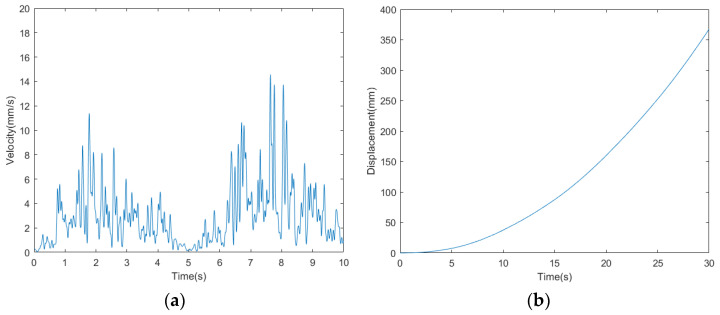
(**a**) Walking speed profile of a bionic millipede robot with traveling wave gait. (**b**) Walking displacement curves of a bionic millipede robot with traveling wave gait.

The velocity curve of the bio-inspired millipede robot is depicted in Figure 18. According to the measurement and analysis of experimental results, the average velocity of the robot is determined to be 10.33 mm/s. Within a period of 30 s, the bio-inspired millipede robot platform traversed a distance of 310 mm, demonstrating its ability to maintain a relatively stable walking speed.

#### 5.3.2. Walk with Broken Legs

**Figure 19:**
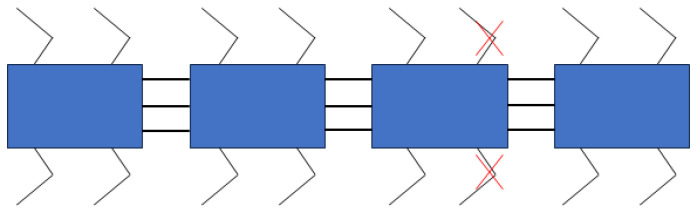
Schematic diagram of the millipede robot walking with broken legs.

The purpose of this section’s experiment is to simulate the scenario where a robot experiences a malfunction during walking, leading to the inability of one or more legs to operate. Based on the physiological study of the biological millipede, it is known that millipedes exhibit high robustness, and they can continue to walk normally even when one or more legs are damaged. Therefore, this section aims to experimentally verify whether the robot can still walk normally after disconnecting the signals to two servo motors. The schematic diagram of disconnecting the signal servo motors is shown in Figure 19.

**Figure 20:**
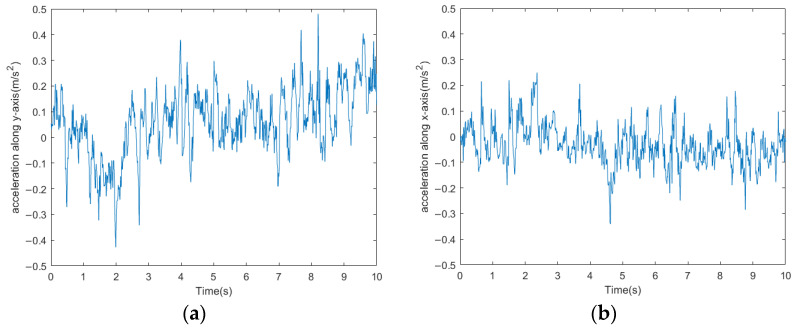
(**a**) Longitudinal acceleration curves for walking with broken legs of the millipede robot. (**b**) Lateral acceleration curves for walking with broken legs of the millipede robot.

**Figure 21:**
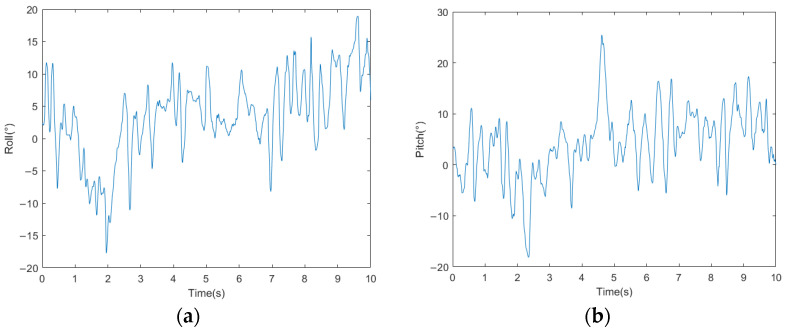
(**a**) Millipede robot walking with broken legs *x*-axis attitude angle. (**b**) Millipede robot walking with broken legs *y*-axis attitude angle.

**Figure 22:**
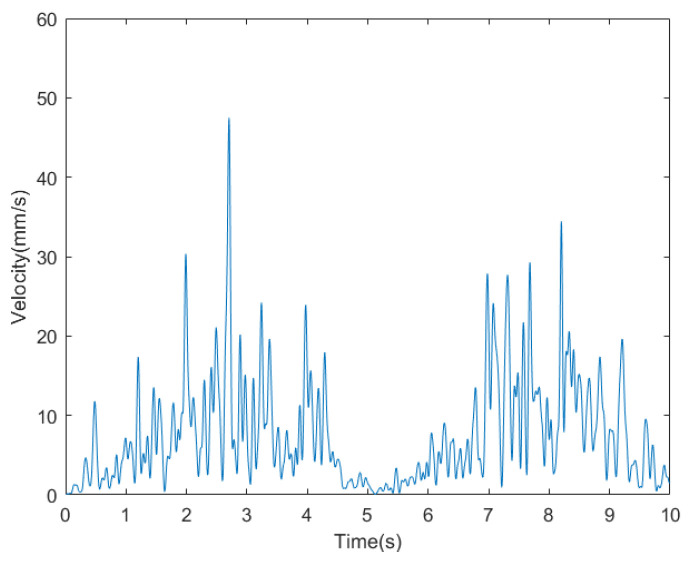
Walking speed profile of the millipede robot with broken legs.

The acceleration curve and attitude angle curve measured by the attitude sensor are shown in Figure 20 and Figure 21, respectively. The velocity curve obtained from the frequency domain transformation of the acceleration data is shown in Figure 22. From the figures, it can be observed that after disconnecting the servo motor signals as shown in Figure 16, the fluctuations in acceleration, attitude angles, and velocity of the millipede robot are similar to those during normal walking. However, it is evident from the lateral acceleration curve, *Y*-axis attitude angle curve, and velocity curve that there are significant fluctuations at certain moments during walking. These fluctuations occur because some servo motors, which should have been disconnected, are still attempting to move the legs. Despite these fluctuations, the robot is still able to walk relatively normally. Therefore, the robot platform designed in this study also demonstrates a certain level of robustness.

#### 5.3.3. Walking on Complex Surfaces

**Figure 23:**
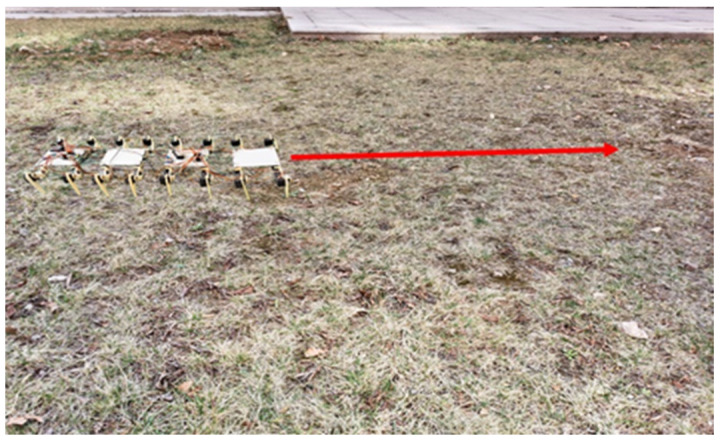
Experimental diagram of the millipede robot walking on complex road surface.

In this section, experiments were conducted to test the walking capabilities of the biomimetic millipede robot on complex terrain. The experiments were performed on the complex terrain depicted in Figure 23, with the red arrow indicating the direction of travel. The purpose was to verify whether the designed millipede robot has the ability to walk on complex terrain.

**Figure 24:**
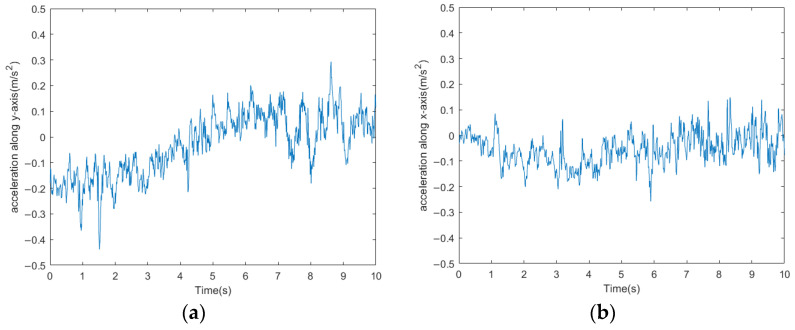
(**a**) Longitudinal acceleration curve for the millipede robot walking on complex road surfaces. (**b**) Lateral acceleration curve of the millipede robot walking on complex road surface.

**Figure 25:**
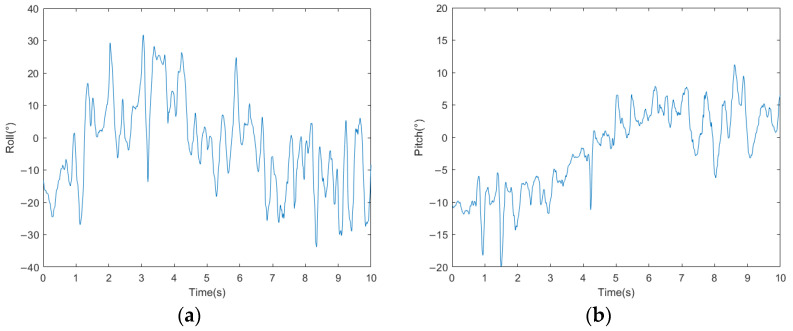
(**a**) The *x*-axis attitude angle curve of the millipede robot walking on a complex road surface. (**b**) The *y*-axis attitude angle curve of the millipede robot walking on a complex road surface.

The millipede robot moves in the direction indicated by the red arrow on the complex terrain, and its acceleration curve is shown in Figure 24, while the attitude angle curve is depicted in Figure 25. 

From the acceleration and attitude angle curves, it can be observed that the millipede robot experiences the influence of the irregularities on the terrain while walking on the complex surface. The fluctuations in both longitudinal and lateral accelerations are greater compared to walking on a hard surface, with the longitudinal acceleration fluctuation increasing to 0.4 m/s^2^ and the lateral acceleration fluctuation increasing to 0.5 m/s^2^. Moreover, the *X*-axis and *Y*-axis attitude angles of the robot’s body also increase compared to walking on a hard surface, especially the *Y*-axis attitude angle, which increases by about 10°. Despite the increased fluctuations compared to walking on a hard surface, the platform can still walk relatively steadily.

**Figure 26:**
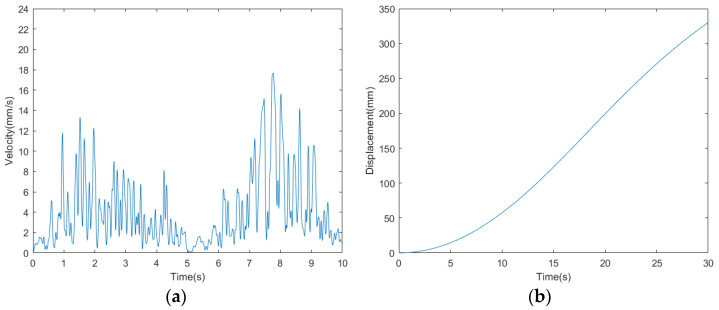
(**a**) Walking speed curve of the millipede robot with traveling wave gait. (**b**) Walking displacement curve of the millipede robot with traveling wave gait.

The speed and displacement curves of the millipede robot walking on complex terrain are illustrated in Figure 26. According to the measurement and analysis of the experimental results, it is determined that the average speed of the millipede robot walking on complex terrain is 11 mm/s. Within 30 s, it covers a distance of approximately 330 mm. This represents a 10.78% reduction in speed compared to walking on a hard surface, indicating a relatively minor decrease in speed. Thus, it is demonstrated that walking on complex terrain can still maintain its walking speed.

Observing the velocity curves of the three sets of experiments above, it can be observed that around the fifth second, the velocity curves of all three experiments approach zero. This occurs because the gait cycle of the millipede robot is approximately 5 s. At around 5 s, its legs have completed one cycle of movement and are preparing to start the next cycle, which results in a brief pause of the millipede robot’s body. This temporary pause leads to the velocity approaching zero around the 5th second.

## 6. Conclusions

In this study, we have systematically investigated the locomotion mechanism and biological characteristics of the millipede as the biomimetic prototype. Our research outcome culminated in the successful development of a prototype of a biomimetic millipede robot, which embodies key features inspired by the biological millipede. Building upon this robot, we conducted in-depth analysis of the kinematics and control algorithms governing its walking legs, effectively validating its locomotive capabilities and stability during motion. Through experimental testing, our biomimetic millipede robot prototype has demonstrated outstanding locomotion capabilities and stability. It can efficiently traverse irregular and rugged terrain, exhibiting remarkable agility and stability. The robot’s body structure and locomotion mechanism closely resemble those of real millipede organisms, enabling it to flexibly navigate various complex environments and obstacles.

1. In studies of millipede’s wave gait, single-leg reference circle or ellipse models are commonly used, but ensuring stability of posture is challenging. This paper proposes a general model with three degrees of freedom based on the analysis of millipede physiology and locomotion mechanisms. By implementing forward and inverse kinematics of the leg structure, which consists of three linked rods in series, the effective and controllable variation of leg length is achieved, ensuring smooth motion of the entire robot while also accommodating a planar model at the foot end.
2. The three-degree-of-freedom walking mechanism combined with the matching CPG algorithm offers higher computational efficiency and simpler model construction compared to traditional control methods based on static stability and dynamic model-based control.
3. Modeled after the actual neural structure, the oscillatory coupling framework reduces the number of oscillators by 75%. During locomotion, the fluctuation of longitudinal acceleration is less than 0.2 m/s^2^, and the fluctuation of lateral acceleration is less than 0.3 m/s^2^, ensuring the stability of the walking gait.
4. The ecological analysis of the millipede and its corresponding locomotion system can be applied not only to conventional hard surfaces; they can also be further optimized to adapt to harsh environments such as farmland slopes, tidal flats, deserts, and barren wastelands. By incorporating various attachments, the millipede robot can achieve functions including load-bearing, operation, rescue, and detection.

## Figures and Tables

**Table 1:** D-H parameter table for the legs of the biomimetic millipede robot.

i	α*_i_*_−1_	*a_i_*_−1_	*d_i_*	*θ_i_*
1	0	0	0	*θ*_c_
2	90	*l*_c_	0	*θ*_f_
3	0	*l*_f_	0	*θ*_t_
4	0	*l*_t_	0	0

**Table 2:** Table of main structural parameters of bionic millipede robot prototype.

Parameters	Numerical Value
size of body segments	190 mm × 150 mm × 6 mm
length of first connecting rod	110 mm
length of second connecting rod	95 mm
length of third connecting rod	130 mm
hip angle	−30–30°
femur and tibia angle	−45–45°
spring length	120 mm
spring stiffness	1.3 N/mm
weight	8 kg

## Data Availability

The data used to support the findings of this study are included within the article.

## References

[1] Fan J., Du Q., Dong Z., Zhao J., Xu T. (2022). Design of the Jump Mechanism for a Biomimetic Robotic Frog. Biomimetics.

[2] Chen G., Qiao L., Zhou Z., Richter L., Ji A. (2023). Development of a Lizard-Inspired Robot for Mars Surface Exploration. Biomimetics.

[3] Pei X., Liu S., Wei A., Shi R., Dai Z. (2023). Bioinspired Rigid–Flexible Coupled Adaptive Compliant Motion Control of Robot Gecko for Space Stations. Biomimetics.

[4] Goldfarb M., Gogola M., Fischer G., Garcia E. (2001). Development of a piezoelectrically-actuated mesoscale robot quadruped. J. Micromechatron..

[5] Birkmeyer P., Peterson K., Fearing R.S. DASH: A dynamic 16g hexapedal robot. Proceedings of the 2009 IEEE/RSJ International Conference on Intelligent Robots and Systems.

[6] Ho T., Choi S., Lee S. (2007). Development of a biomimetic quadruped robot. J. Bionic Eng..

[7] Ni Y., Li L., Qiu J., Sun Y., Qin G., Han Q., Ji A. (2022). A Novel Wheel-Legged Hexapod Robot. Biomimetics.

[8] Zarrouk D., Mann M., Degani N., Yehuda T., Jarbi N., Hess A. (2016). Single actuator wave-like robot (SAW): Design, modeling, and experiments. Bioinspir. Biomim..

[9] Mingchinda N., Jaiton V., Leung B., Manoonpong P. (2023). Leg-body coordination strategies for obstacle avoidance and narrow space navigation of multi-segmented, legged robots. Front. Neurorobot..

[10] Garcia A., Priya S., Marek P. Understanding the locomotion and dynamic controls for millipedes: Part 1—Kinematic analysis of millipede movements. Proceedings of the Smart Materials, Adaptive Structures and Intelligent Systems.

[11] Manton S. (1952). The evolution of arthropodan locomotory mechanisms—Part 3. the locomotion of the chilopoda and pauropoda. Zool. J. Linn. Soc..

[12] Homchanthanakul J., Manoonpong P. (2023). Proactive body joint adaptation for energy-efficient locomotion of bio-inspired multi-segmented robots. IEEE Robot. Autom. Lett..

[13] Wan X., Song S.-M. A cam-controlled, single actuator-driven leg mechanism for legged vehicles. Proceedings of the ASME International Mechanical Engineering Congress and Exposition.

[14] Garcia A., Krummel G., Priya S. (2020). Fundamental understanding of millipede morphology and locomotion dynamics. Bioinspir. Biomim..

[15] Kano T., Sakai K., Yasui K., Owaki D., Ishiguro A. (2017). Decentralized control mechanism underlying interlimb coordination of millipedes. Bioinspir. Biomim..

[16] Koh D., Yang J., Kim S. Millipede robot for uneven terrain exploration: Design and experiment of the flexible biomimetic robot mechanism. Proceedings of the 2010 3rd IEEE RAS & EMBS International Conference on Biomedical Robotics and Biomechatronics.

[17] Ozkan-Aydin Y., Chong B., Aydin E., Goldman D.I. A systematic approach to creating terrain-capable hybrid soft/hard myriapod robots. Proceedings of the 2020 3rd IEEE International Conference on Soft Robotics (RoboSoft).

[18] Hoffman K.L. (2013). Design and Locomotion Studies of a Miniature Millipede-Inspired Robot. Doctoral Dissertation.

[19] Avirovik D., Butenhoff B., Priya S. (2014). Millipede-inspired locomotion through novel U-shaped piezoelectric motors. Smart Mater. Struct..

[20] Avirovik D., Priya S. Crawling-inspired robot utilizing L-shape piezoelectric actuators. Proceedings of the 2013 IEEE/ASME International Conference on Advanced Intelligent Mechatronics.

[21] Brown T.G. (1911). The intrinsic factors in the act of progression in the mammal. Proc. R. Soc. Lond. B.

[22] Matsuoka K. (1987). Mechanisms of frequency and pattern control in the neural rhythm generators. Biol. Cybern..

[23] Kimura H., Fukuoka Y., Cohen A.H. (2007). Adaptive Dynamic Walking of a Quadruped Robot on Natural Ground Based on Biological Concepts. Int. J. Robot. Res..

[24] Righetti L., Ijspeert A.J. Design methodologies for central pattern generators: An application to crawling humanoids. Proceedings of the Robotics: Science and Systems.

[25] Ijspeert A.J., Crespi A., Ryczko D., Cabelguen J.-M. (2007). From swimming to walking with a salamander robot driven by a spinal cord model. Science.

[26] Grabowska M., Toth T.I., Smarandache-Wellmann C., Daun-Gruhn S. (2015). A network model comprising 4 segmental, interconnected ganglia, and its application to simulate multi-legged locomotion in crustaceans. J. Comput. Neurosci..

[27] Song Z., Zhu J., Xu J. (2023). Gaits generation of quadruped locomotion for the CPG controller by the delay-coupled VDP oscillators. Nonlinear Dyn..

[28] Chen L., Cai Y., Bi S. (2023). Central Pattern Generator (CPG)-Based Locomotion Control and Hydrodynamic Experiments of Synergistical Interaction between Pectoral Fins and Caudal Fin for Boxfish-like Robot. Biomimetics.

[29] Asif U. (2012). Virtual Reality to Simulate Adaptive Walking in Unstructured Terrains for Multi-Legged Robots. Virtual Real. Hum. Comput. Interact..

[30] Yu J., Tan M., Chen J., Zhang J. (2013). A survey on CPG-inspired control models and system implementation. IEEE Trans. Neural Netw. Learn. Syst..

[31] Wang Z., Gao Q., Zhao H. (2017). CPG-inspired locomotion control for a snake robot basing on nonlinear oscillators. J. Intell. Robot. Syst..

[32] Nor N.M., Ma S. (2014). A simplified CPGs network with phase oscillator model for locomotion control of a snake-like robot. J. Intell. Robot. Syst..

[33] Righetti L., Ijspeert A.J. Pattern generators with sensory feedback for the control of quadruped locomotion. Proceedings of the 2008 IEEE International Conference on Robotics and Automation.

